# A Retrospective Longitudinal Study in a Cohort of Children With Dyskinetic Cerebral Palsy Treated With Tetrabenazine

**DOI:** 10.3389/fneur.2021.612429

**Published:** 2021-02-26

**Authors:** Roberta Scalise, Giuseppina Sgandurra, Valentina Menici, Nicola Capodagli, Roberta Di Pietro, Domenico M. Romeo, Francesca Sini, Emanuela Pagliano, Maria Foscan, Giovanni Cioni, Roberta Battini

**Affiliations:** ^1^Department of Developmental Neuroscience, IRCCS Fondazione Stella Maris, Pisa, Italy; ^2^Tuscan PhD Programme of Neuroscience, University of Florence, Pisa and Siena, Florence, Italy; ^3^Department of Clinical and Experimental Medicine, University of Pisa, Pisa, Italy; ^4^Pediatric Neurology Unit, Catholic University, Fondazione Policlinico A. Gemelli, IRCCS, Rome, Italy; ^5^Neurodevelopmental Unit, Fondazione IRCCS Istituto Neurologico “Carlo Besta”, Milan, Italy

**Keywords:** dyskinetic cerebral palsy, tetrabenazine, children, MD-CRS 4–18 R, movement disorder

## Abstract

Tetrabenazine has been studied with a variety of hyperkinetic movement disorders, but there is limited and empirical literature on the potential efficacy of tetrabenazine in children with dyskinetic cerebral palsy (DCP). The purpose of this study was to evaluate the efficacy of tetrabenazine in a sample of children with DCP using the Movement Disorders—Childhood Rating Scale 4–18 Revised (MD-CRS 4–18 R). The study is a multicenter retrospective longitudinal study in which the participants were selected from the databases of each Center involved, according to detailed inclusion criteria. The study was performed on 23 children and adolescents (19 male and 4 females; mean age 8.28 years, SD 3.59) with DCP having been evaluated before starting the treatment (baseline), after 6 and 12 months of treatment and in a sub-cohort after >2 years follow-up. A linear mixed model was used to evaluate the effects of the different timings on each MD-CRS 4–18 R Index (Index I, Index II, and Global Index) adding age and type of movement disorder as random effect. A significant clinical improvement related to a reduction of MD-CRS 4-18 R Indexes was detected between the baseline and after 6 and 12 months of treatment. Findings support the efficacy of tetrabenazine in children with DCP through a standardized outcome measure (MD-CRS 4–18 R) and confirm the use of this scale as a suitable tool to detect changes in further randomized clinical trials.

## Introduction

Tetrabenazine (TBZ; Xenazine®) is a selective and reversible depletor of monoamines from synaptic terminals, preferentially dopamine but also norepinephrine, serotonin, histamine (1). TBZ has a short half-life, which lasts for about 16–24 h, and a rapid onset of action, which is useful in clinical applications (2). The antichorea efficacy of TBZ is related to dopamine depletion, whereas the risk of depression may be mediated by serotonin and norepinephrine depletion, and sedation may be explained by histamine depletion (1).

TBZ could determine a mild increase in the corrected QT (QTc) interval and should be used properly and carefully when combined with other drugs that also increase the interval of QTc or in patients with congenital long QT syndrome and a history of cardiac arrhythmias (3).

The dose of TBZ should be personalized for each patient (3), with significantly interindividual differences in reaching the “optimal dose,” which is the dose that provides the main clinical response with minimal or tolerable adverse events (1).

The principal most common and dose-limiting side effects of TBZ are known. These include sedation (28%), akathisia (13%), parkinsonism (7%), depression (5.5%), anxiety (4%), fatigue (2%), and diarrhea (2%), all of which are usually rapidly reversible upon dosage reduction (1, 2, 4).

To date, there is no consensus regarding the best clinical practice in the pediatric population; in this instance, the administration of TBZ to the adult population is empirically adapted to children and it is “off-label” (5) for a wide variety of hyperkinetic movement disorders (6–10), including dyskinetic cerebral palsy (DCP) (11).

DCP, the second most common type of cerebral palsy (CP) (almost 15%), typically caused by non-progressive basal ganglia and/or thalamus lesions, is characterized by abnormal postures or movements associated with compromised tone regulation and coordination. In DCP, the two typical movement disorders are dystonia and choreoathetosis, which are often co-occurring; dystonia is usually more severe than choreoathetosis, affecting daily activity, quality of life, and social participation (12).

A recent review has highlighted the inadequate evidence for pharmacological interventions in DCP due to the absence of any therapeutic algorithm and the lack of reliable, valid, and agreed-upon age-specific outcome measures (11). To fill this gap, in 2008, the Childhood Movement Disorders Rating Scale (MD-CRS) was proposed and has been recently updated in a revised form (MD-CRS 4-18 R). The scale aimed to define the functional impairment and the severity produced by movement disorder (e.g., dystonia and choreoathetosis) in the pediatric population (11, 13–15). The psychometric properties in DCP of MD-CRS 4–18 R have been recently published (15).

In this study, we have hypothesized that (i) the use of standardized outcome measures, such as MD-CRS, could provide useful data for a retrospective analysis of “off-label” treatment with TBZ obtained from routine clinical practice in the pediatric population affected by DCP; (ii) clinical data could provide a preliminary insight for evaluating the safety and effectiveness of TBZ treatment.

The primary purpose of this study was to retrospectively evaluate a sample of children with DCP who had been treated with TBZ in order to collect data on its efficacy using the MD-CRS 4–18 R as a standardized tool to assess movement disorders in childhood and to detect changes during treatment.

The secondary aims of the study were (1) to determine the impact of TBZ-associated side effects in children with DCP and (2) to analyze the long-term effectiveness and tolerability of TBZ in clinical practice, by evaluating the outcome on a sub-group with a follow-up >2 years.

## Materials and Methods

### Study Design

A multicenter retrospective longitudinal study was designed involving three Italian Research and Clinical Scientific Institutes: The Department of Developmental Neuroscience of Stella Maris Foundation (Pisa), The Developmental Neurology Unit Institute “C. Besta” (Milano), and The Pediatric Neurology Unit of Fondazione Policlinico “A. Gemelli” (Rome).

**Figure 1:**
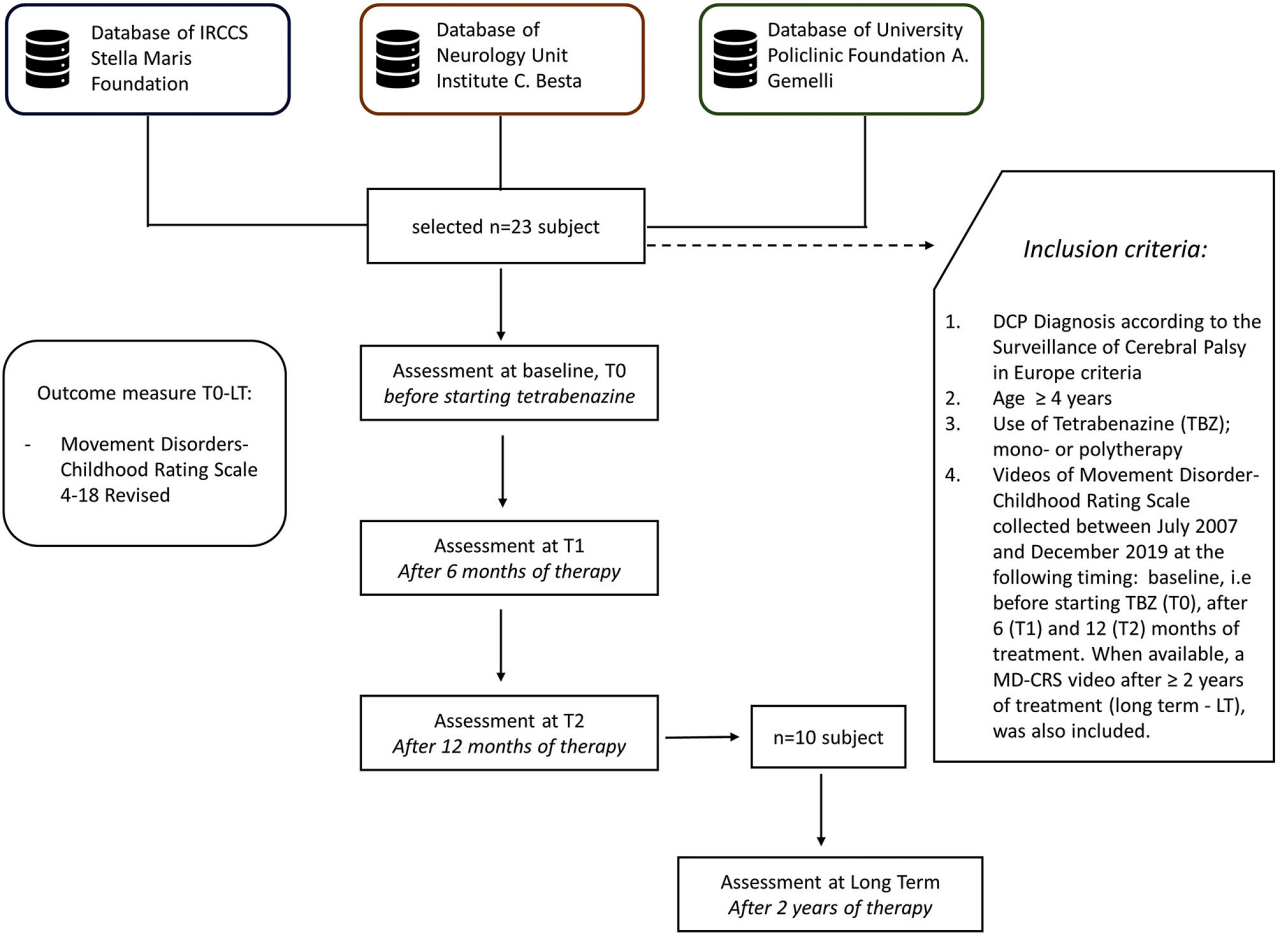
Design and management of the study with subjects selection.

These scientific institutes cooperate in the research on movement disorders, sharing agreed clinical and pharmacological protocols, including outcome measures, as MD-CRS. Each Center has its own database for CP data collection, which accounts for at least 600 subjects. From these data sets, cases were selected according to the following inclusion criteria: (a) DCP diagnosis according to the Surveillance of Cerebral Palsy in Europe criteria (16); (b) age ≥4 years; (c) use of TBZ, in mono- or polytherapy—in the presence of polytherapy a stable dosage of other drugs was maintained during the 12 months of follow up, after starting TBZ; (d) videos of Movement Disorder-Childhood Rating Scale which had been collected between July 2007 and December 2019, at the following timing: baseline, i.e., before starting TBZ (T0), after 6 (T1), and 12 (T2) months of treatment. When available, a MD-CRS video after ≥2 years of treatment (long term—LT), was also included (Figure 1).

### Procedures

The clinical, demographic, and pharmacological data of each selected subject were extracted from medical records to fulfill the aims of the study.

All participants were classified according to the Gross Motor Function Classification System (GMFCS) for cerebral palsy which is based on self-initiated movement, with emphasis on sitting, transfers, and mobility. A five-level classification system is used to define the subject (from I to V increasing severity) and the distinctions are based on functional limitations, the need for hand-held mobility devices or wheeled mobility, and to a much lesser extent, quality of movement.

A neurological and psychiatric history assessment was carried out on all the subjects.

The psychiatric evaluation was carried out by anamnestic interview and, when available for subjects >6 years, with some items from a semi-structured interview, the Schedule for Affective Disorders and Schizophrenia for School-Age Children–Present and Lifetime Version (K-SADS-PL).

These evaluations were checked before and during the follow up visit.

TBZ treatment indications for all the included subjects were based on multidisciplinary assessments made by child neurologists, an expert in movement disorders, and a pediatric physical therapist.

TBZ administration usually started with a low fixed dose of 6.25 mg (1/4 oral tablet of 25 mg) at least during the first week and was gradually and variably increased according to weight or the clinical neuromotor picture with an aim of reaching a “possible optimal dose,” as in previous reports (1, 4). The dosage was subdivided into 2 or 3 times daily.

Data collection of corrected QT (QTc) was considered if the subjects had performed an EKG before the study, during up-titration, and subsequently annually during follow-up to monitor the risk of a potential increase in the QTc interval or cardiac arrhythmias.

### Outcome Measure

All the videos included were recorded according to a standardized videoprotocol and blind scored according to MD-CRS 4–18 R (15).

MD-CRS 4–18 R is a feasible tool to verify the natural history of the disease and represents a standardized clinical outcome measure in the evaluation and follow-up of children with DCP.

The MD-CRS 4–18 R was developed for the assessment of function and disability in various types of movement disorders. This scale requires that the evaluation be recorded according to a specific videoprotocol, and subsequently a score is assigned. The MD-CRS 4–18 R scale is divided into two parts: General Assessment (Part I) and Movement-Disorder Severity (Part II). In Part I, four areas are included: motor function, oral/verbal function, self-care, and attention/alertness, for a total of 15 items. In Part II, the intensity of the prevalent movement abnormality in seven body parts (eye and periorbital region, face, tongue and perioral region, neck, trunk, upper limb, lower limb) in two conditions, at rest and during the execution of specific tasks, is assessed. All items are scored on a 5-point ordinal scale (0–4): zero corresponds to no signs, and 4 corresponds to the most severe findings. The scores for part I and part II and the total score are calculated by statistical analysis obtaining Index I, Index II, and Global Index, respectively. Indexes range come from 0 (severe impairment) to 1 (normal).

The *Stella Maris Foundation* holds the copyright for the MD-CRS 4–18 R (MD-CRS copyright owner) which is distributed, worldwide, by Mapi Research Trust, a nonprofit organization. For further information and conditions of use of the MD-CRS 4–18 R, consult the online platform Mapi Research Trust, ePROVIDE (https://eprovide.mapi-trust.org).

### Ethics Approval

The multicenter study was approved by the Tuscany Pediatric Ethics Committee (94/2019). All the parents were asked to sign an informed written consent form for treatment “off label” at baseline.

### Statistical Analyses

A linear mixed model was used to evaluate the effects of the different timings (T0, T1, and T2 as fixed factors) on each Index (Index I, Index II, and Global Index) adding age and type of MD as random effect. Further analysis with the same approach was also carried out for the subgroup of subjects with the long-term follow-up to evaluate the effects of T0, T1, T2, and LT on each index. For both models, a *post hoc* analysis between different time points was performed.

## Results

### Participants

The study included 23 children and adolescents (19 male and 4 female) affected by DCP, age range 4.02–16.30 years at baseline observation (mean age 8.28 years, SD 3.59).

Clinical and demographic data are reported in Table 1.

**Table 1:** Clinical and demographic data of the sample.

**Demographic**	**Participants *N* = 23**
Sex: n (%)	Male: 19 (83%) Female 4 (17%)
Mean age ± SD (range) at T0 (years)	8.28 ± 3.59 (4.02–16.30)
Mean weight ± SD (range) at T0 (kg)	25.13 ± 11.94 (12.00–62.00)
Type of MD: n (%)	Dystonia and choreoathetosis: 19 (83%) Choreoathetosis: 4 (17%)
Etiology: n (%)	Hypoxic-ischemic encephalopathy: 17 (74%) Kernicterus: 4 (17%) Cytomegalovirus infection: 2 (9%)
GMFCS level: n (%)	I: 1 (4%) II: 1 (4%) III: 2 (9%) IV: 5 (22%) V: 14 (61%)

None of the participants presented clinically relevant psychiatric diseases (e.g., significant depression or history of suicidal intent) at baseline (T0).

At the beginning of TBZ treatment, seven subjects with DCP started TBZ in monotherapy, while the others were in polytherapy (e.g., oral baclofen, trihexyphenidyl, benzodiazepines).

A mean dosage of TBZ close to 1 mg/kg was reached (Table 2).

**Table 2:** Mean and range TBZ dosage at the different time points.

**Timing**	**Mean (mg/kg) ± SD**	**Min (mg/kg)**	**Max (mg/kg)**
T1 (T0 +6 months)	0.70 ± 0.41	0.30	2.08
T2 (T0 +12 months)	0.84 ± 0.44	0.20	2.08
Long Term	0.97 ± 0.58	0.20	2.08

*T0, baseline; T1, 6 months of therapy; T2, 12 months of therapy; LT, long term, > 2 year of therapy*.

The reduction of the minimum dosage after 12 months and at long term after treatment was related to the maintenance of a stable oral dose between T1 and T2 (5 subjects) without considering the weight increase of the subjects. 4 of the other children reduced their dosage, 2 after 6 months, and 2 after 12 months respectively, due to the occurrence of adverse events (see specific section).

### Clinical Outcomes at Baseline (T0), After 6 (T1), and 12 Months (T2) of TBZ Treatment

A relevant effect of timing was found with a significant reduction of each index between T0 and T1 and between T0 and T2, but not between T1 and T2. Specifically, the mean value of Index I (General Assessment) at T0 0.60 ± 0.06, at T1 0.57 ± 0.06, and at T2 0.57 ± 0.06; the mean value for Index II (MD severity) at T0 was 0.63 ± 0.03, at T1 0.53 ± 0.03, and at T2 0.48 ± 0.03; finally, the mean value of for Global Index at T0 was 0.61 ± 0.05, at T1 0.56 ± 0.05, and at T2 0.54 ± 0.05.

The results of the analysis at T0, T1, and T2 are reported in Table 3.

**Table 3:** Linear mixed model results during 12 months of follow up.

**MD-CRS 4–18 R indexes**	**Timing**	**Coefficient estimate (SD)**	***p*-value**	**R^**2**^**
Index I	Intercept	0.60 (0.06)	>0.05	0.98
	T1	−0.03 (0.01)	<0.01	
	T2	−0.03 (0.01)	<0.01	
Index II	Intercept	0.63 (0.03)	<0.01	0.73
	T1	−0.10 (0.03)	<0.01	
	T2	−0.15 (0.03)	<0.01	
Global index	Intercept	0.61 (0.05)	>0.05	0.95
	T1	−0.05 (0.01)	<0.01	
	T2	−0.07 (0.01)	<0.01	

*MD-CRS R, movement disorders—childhood rating scale revised; T0, baseline; T1, 6 months of therapy; T2, 12 months of therapy*.

As shown, a significant effect of timing was found at T1 and T2 (Table 3).

### Clinical Outcomes at Baseline (T0), After 6 (T1), and 12 Months (T2) and TBZ Long-Term (LT) Treatment

A small group, 10 subjects, had a long-term follow-up >2 years, and the efficacy of TBZ was still present and stable without important side effects.

In this long-term group (LT), a significant reduction of each Index was found between T0 and T2 but not between T2 and LT. Specifically, the MD-CRS R Index I (General Assessment) mean value was 0.52 ± 0.08 at T0, 0.48 ± 0.08 at T1, 0.49 ± 0.08 at T2, and 0.46 ± 0.08 at long term; the Index II (MD Severity) mean value was 0.62 ± 0.06 at T0, 0.52 ± 0.06 at T1, 0.49 ± 0.06 at T2, and 0.52 ± 0.06 at long term; the Global Index mean value was 0.55 ± 0.07 at T0, 0.49 ± 0.07 at T1, 0.48 ± 0.07 at T2, and 0.48 at long term.

The *post hoc* analysis between the different time points during 12 months follow up are reported in Table 4. The results of the analysis on the T0, T1, T2, and long-term subgroup are reported in Table 5. As shown, all-time points are significant (Table 5).

**Table 4:** *Post-hoc* analysis between the different time points during 12 months follow up.

**Timing**	**MD-CRS 4-18 R indexes**	**Estimate (SE)**	**Degrees of freedom**	**t ratio**	***p*-value**
T0–T1	Index I	0.03 (0.01)	42	2.92	<0.05
	Index II	0.10 (0.02)	42	3.81	<0.01
	Global index	0.05 (0.01)	42	4.15	<0.01
T0–T2	Index I	0.03 (0.01)	42	3.06	<0.05
	Index II	0.15 (0.03)	42.3	5.38	<0.01
	Global index	0.07 (0.01)	42	5.40	<0.01
T1–T2	Index I	0.01 (0.01)	42	0.24	> 0.05
	Index II	0.05 (0.02)	42.3	1.69	> 0.05
	Global index	0.02 (0.01)	42	1.39	> 0.05

*MD-CRS 4–18 R, movement disorders—childhood rating scale 4–18 revised; T0, baseline; T1, 6 months of therapy; T2, 12 months of therapy*.

**Table 5:** Linear mixed model results during the long-term follow-up.

**MD-CRS 4–18 R indexes**	**Timing**	**Coefficient estimate (SD)**	***p*-value**	**R^**2**^**
Index I	Intercept	0.52 (0.08)	<0.01	0.98
	T1	−0.05 (0.01)	<0.01	
	T2	−0.03 (0.01)	<0.05	
	LT	−0.07 (0.02)	<0.01	
Index II	Intercept	0.62 (0.06)	<0.01	0.75
	T1	−0.10 (0.04)	<0.05	
	T2	−0.13 (0.04)	<0.05	
	LT	−0.09 (0.04)	<0.05	
Global index	Intercept	0.55 (0.07)	<0.01	0.96
	T1	−0.06 (0.02)	<0.01	
	T2	−0.07 (0.02)	<0.01	
	LT	−0.08 (0.02)	<0.01	

*MD-CRS 4–18 R, movement disorders—childhood rating scale 4–18 revised; T0, baseline; T1, 6 months of therapy; T2, 12 months of therapy; LT, long term, >2 year of therapy*.

In the following table, the result of the *post-hoc* analysis of long-term vs. baseline (T0) and T2 are represented (Table 6).

**Table 6:** *Post-hoc* analysis between the different time points during the long-term follow-up.

**Timing**	**MD-CRS 4–18 R indexes**	**Estimate (SE)**	**Degrees of freedom**	**t ratio**	***p*-value**
LT–T0	Index I	−0.07 (0.02)	28	−4.44	<0.01
	Index II	−0.09 (0.04)	28.2	−2.13	<0.05
	Global index	−0.08 (0.02)	28	−3.78	<0.01
LT–T2	Index I	−0.03 (0.01)	28	−2.22	<0.05
	Index II	0.04 (0.04)	28	0.79	> 0.05
	Global index	−0.01 (0.02)	28	−0.22	> 0.05

*MD-CRS 4–18 R: movement disorders—childhood rating scale 4–18 revised; t0, baseline; t2, 12 months of therapy; lt, long term, >2 year of therapy*.

### TBZ-Associated Side Effects

TBZ was associated with side effects in 6/23 (26%) subjects: drowsiness in 3/23 (13.04%); dystonia in 2/23 (8.69%); parkinsonism/rigidity in 2/23 (8.69%), anxiety and depressive problems in 1 (4.35%), and akathisia in 1 (4.35%).

Three of these children showed two side effects: one child showed drowsiness after 6 months of treatment and parkinsonism/rigidity after 12 months; another showed dystonia after 6 months of therapy and anxious-depressive problems in the long-term follow-up while the third child presented dystonia and akathisia after 12 months of therapy. Of these subjects, 2 dropped out after T1, one due to dystonia and the other to parkinsonism/rigidity which was treated subsequently with the implantation of Intrathecal Baclofen Pump.

An ECG (included QTc) was performed on all individuals with QTc at baseline (T0) and during up-titration, T1, and T2. The QTc value remained in a normal range in all subjects. Two children reported no clinically significant tachycardia.

## Discussion

To our knowledge, this is the first study dedicated to a homogeneous cohort of children with DCP treated with TBZ who were subsequently followed up for at least 1 year and evaluated with a standardized clinical outcome (MD-CRS). Specifically, it represents the results of a routine medical practice by monitoring, by a standardized outcome measure, the use of TBZ when prescribed to children with DCP.

In this study, the use of TBZ was determined by the ineffectiveness of other dystonia and chorea drugs or it was prescribed as an add-on to hyperkinetic movement disorder treatment specifically. The effects of TBZ in children with DCP, either in a dystonic or choreic form, were evaluated, and a clinical improvement was reported regarding both the severity and quantity of the movement disorder in different parts of the body. Improvement in general clinical functioning, such as the motor and self-care abilities and activities, was also detected.

During the titration period, the dose of TBZ was adjusted empirically according to the clinical benefit: the starting dose of 6.25 mg (1/4 oral tablet of 25 mg) was prescribed once daily, and it was titrated gradually increasing it to 6.25 mg weekly or bi-weekly until a maximum of 50 mg/day (2 mg/kg, twice or three times daily).

In order to identify a dose level that clinically reduced movement disorders and was well tolerated, a customized TBZ dose regimen was considered for each subject. This step is a very crucial achievement because it shows the common behavior of clinicians to customize the TBZ dose on the basis of the clinical improvement and functioning. In general, research impacts the clinical behavior, but in this case, as is important in evidence-based medicine (EBM), clinical expertise is essential and works in parallel with research evidence and patient preferences for guiding the EBM.

The data obtained by clinical observation were considered because there is no consensus in literature regarding the optimal medical treatment currently available (11), and the therapies are mainly related to “off-label” drugs anecdotally adapted to children (17).

With regard to the pediatric population, some authors, already many years ago, used a higher initial drug schedule: for age 3–10 years, the final dose was 25 mg twice a day, and for age >10 years the final dose was 50 mg twice a day, but due to the occurrence of drowsiness, the drug regimen was reduced to half dose (18). Other studies (5, 7, 19–21) on the use of TBZ treatment in the hyperkinetic movements, but not related to DCP or children, introduce a wide range of doses of TBZ which vary from 12.5 to 350 mg/day based on the positive drug response.

These findings were confirmed in the current study: in common clinical practice, the optimal dose of TBZ in the treatment of DCP in a pediatric population is individualized and not related to any specific dose per body weight but according to the clinical improvement.

This study, differently from those undertaken previously, also evaluated the efficacy of TBZ in children with DCP by using MD-CRS 4–18 R, a standardized and suitable tool to detect changes during pharmacological treatment.

Indeed, a significant clinical reduction of movement disorder after 6 months was detected, subsequently followed by a “plateau state.” A significant improvement in the general assessment (including Motor function, Oral/verbal function, Self-care and Attention/Alertness) and in the severity of movement disorders (including grading and spread of movement disorders for different body regions) was found after 6 and 12 months of TBZ treatment. No relevant differences were obtained during TBZ administration between 6 and 12 months. These results are crucial from a clinical point of view because they show how the children and parents can be assured that the use of TBZ impacts not only the reduction of movement disorder but also the clinical general functioning such as language and the self-care activities.

No relevant differences were observed in the type and frequency of adverse events reported in scientific literature and this study. The main side effect, in fact, was sedation, followed by dystonia-parkinsonism, depression, and akathisia.

In the long-term subgroup of the sample, a stable improvement was reached after a treatment period of more than 2 years and no important side effects were detected.

Although no side effects relating to heart rhythm abnormalities were detected, according to our experience, EKG evaluations, including QTc measurement, are advisable before starting TBZ, during up-titration (10 days after each dose increasing) and subsequently an annual follow-up.

The main limitations of this study are related to the small size of the sample and the low dosage of TBZ prescribed, compared to the previous reports. A retrospective design based on the review of each Center database and the inclusion criteria (i.e., polytherapy with stable dosage for at least 12 months during TBZ treatment) were probably responsible for the small size of the cohort and the concomitant use of other medications, which could have interfered with the obtained benefits. In addition, the lack of further clinical improvement after the initial reduction of hyperkinetic movements and the absence of severe side effects could be justified by the low dose used in the study cohort.

However, the present study reflects the common clinical practice in the management of an “off-label” drug in children while at the same time embedding valid and agreed-upon age-specific outcome measures.

In conclusion, this study shows that (a) the efficacy of TBZ in children with DCP through a gradual titration has been demonstrated when a standardized outcome measure is used; (b) an optimal TBZ dose should be individualized according to the clinical improvement, such as the reduction of movement disorders; (c) a standardized outcome measure, such as MD-CRS 4–18 R, is also essential during the clinical observational trial and not only in the randomized trial, since it is important to detect changes in an objective way; and (d) good safety and tolerability of TBZ in children has been proved while the occurrence of the side effects should be evaluated through a clinical neurological and psychiatric follow-up and periodic EKG monitoring.

Further studies, however, are needed in children with DCP by randomized clinical trials including parallel groups with different doses of the drug to determine the standardized dosage to be shared in clinical and research settings.

## Data Availability Statement

The datasets presented in this article are not readily available because the they contain information that could compromise the privacy of the participants. Requests to access the datasets should be directed to rbattini@fsm.unipi.it.

## Ethics Statement

The research was conducted in accordance with Good Clinical Practice, the Declaration of Helsinki and local laws. The study was approved by the Tuscany Pediatric Ethics Committee (94/2019). All the parents were asked to sign an informed written consent form for the treatment “off label” with tetrabenazine.

## Author Contributions

RS contributed to the study concept and design and to the execution of the research project and wrote and edited the first draft of the manuscript. GS contributed to the study concept and design, execution and review of the statistical analysis, and manuscript revision. VM organized the database for data collection, generated the tables, and edited the manuscript. NC proofread the data and wrote a part of the manuscript. DR, FS, RD, EP, and MF contributed to the execution of the research project and data collection. GC read, critically revised, and approved the final manuscript. RB conceived the study concept and design, the execution of the research project, interpretation of results, and critical revision of the submitted version. All authors contributed to the article and approved the submitted version.

## Conflict of Interest

The authors declare that the research was conducted in the absence of any commercial or financial relationships that could be construed as a potential conflict of interest.
